# Development and Characterization of a Primary Ciliated Porcine Airway Model for the Evaluation of In Vitro Mucociliary Clearance and Mucosal Drug Delivery

**DOI:** 10.3390/pharmaceutics17040462

**Published:** 2025-04-02

**Authors:** Janik Martin, Veronika Neubauer, Rebecca Rittersberger, Simon Treitler, Patrick Kopp, Cemre Günday, Iman Shrimo, Annabelle Dabbars, Frank Rosenau, Akif Emre Türeli, Nazende Günday-Türeli, Oliver Haedicke-Peters, Katharina Schindowski

**Affiliations:** 1 Institute of Applied Biotechnology, University of Applied Science Biberach, Hubertus-Liebrecht-Strasse 35, 88400 Biberach, Germany; martin@hochschule-bc.de (J.M.); rittersberger@hochschule-bc.de (R.R.); imanshrimo@gmail.com (I.S.); peters@hochschule-bc.de (O.H.-P.); 2 Faculty of Natural Science, University of Ulm, Albert-Einstein-Allee 11, 89081 Ulm, Germany; 3 MyBiotech GmbH, Industriestraße 1 B, 66802 Überherrn, Germany; c.guenday@mybiotech.de (C.G.); e.tuereli@mybiotech.de (A.E.T.); n.guenday-tuereli@mybiotech.de (N.G.-T.); 4 Institute of Pharmaceutical Biotechnology, Ulm University, Albert-Einstein-Allee 11, 89081 Ulm, Germany; frank.rosenau@uni-ulm.de

**Keywords:** epithelial airway model, inhaled drug delivery, cilia, biopharmaceutics, monoclonal antibodies, therapeutic antibodies, primary cell culture

## Abstract

**Background/Objectives**: In vitro models play a crucial role in preclinical respiratory research, enabling the testing and screening of mucosal formulations, dosage forms, and inhaled drugs. Mucociliary clearance (MCC) is an essential defense mechanism in mucosal drug delivery but is often impaired in respiratory diseases. Despite its importance, standardized in vitro MCC assays are rarely reported. Furthermore, many published methods primarily measure cilia beat frequency (CBF), which requires high-speed cameras that are not accessible to all laboratories. Therefore, this study aimed to develop a physiologically relevant, differentiated in vitro model of the respiratory epithelium that incorporates both beating cilia and functional MCC. We chose porcine airway mucosa as an alternative to human tissue due to ethical considerations and limited availability. The established model is designed to provide a reproducible and accessible method for a broad range of research laboratories. **Methods**: The previously published tracheal mucosal primary cell (TMPC DS) model, derived from porcine tissue, lacked the presence of beating cilia, which are crucial for effective MCC analysis. For accurate MCC assessment, beating cilia are essential as they play a key role in mucus clearance. To address this limitation, the here-described ciliated tracheal mucosal primary cell (cTMPC) model was developed. cTMPCs were isolated from porcine tissue and cultured under air–liquid interface (ALI) conditions for 21 days to promote differentiation. This model was evaluated for cell morphology, tight junction formation, ciliated and mucus-producing cells, barrier function, gene expression, and tracer/IgG transport. MCC and the model’s suitability for standardized MCC assays were assessed using an inverted microscope. In contrast to the TMPC DS model, which lacked beating cilia and thus could not support MCC analysis, the cTMPC model allows for comprehensive MCC studies. **Results**: The developed differentiated in vitro model demonstrated key structural and functional features of the respiratory epithelium, including well-differentiated cell morphology, tight junction integrity, ciliated and mucus-producing cells, and effective barrier function. Functional MCC was observed, confirming the model’s potential for standardized clearance assays. **Conclusions**: This differentiated in vitro model closely replicates the structural and functional characteristics of in vivo airways. It provides a valuable platform for studying mucociliary clearance, toxicology, drug uptake, and evaluating mucosal formulations and dosage forms in respiratory research.

## 1. Introduction

Differentiated in vitro models are indispensable tools in respiratory research, offering critical insights into the physiology and pathology of the human respiratory system. Inhaled drugs typically deposit in both the upper airways (mouth and oropharynx) and lower airways (trachea, bronchi, and lungs) [1]. The deposition pattern of these (bio-)pharmaceuticals is influenced by factors such as particle or droplet size, inhalation means, breathing pattern, or airway obstruction [2]. At the cellular level, mucociliary clearance (MCC) acts as the primary barrier against inhaled particles, facilitated by the coordinated action of cilia and mucus in the conducting airways. The efficiency of MCC depends on the number, structure, coordination, and movement of the cilia, as well as the composition of the mucus, including its water content and mucin structure [3].

The trachea serves as a vital conduit for air passage to the lungs and plays a pivotal role in defending against inhaled pathogens and particles. Its pseudostratified ciliated epithelium comprises beating cilia, mucus-producing goblet cells, club cells, and basal cells, all of which contribute to its protective functions. The barrier function relies on tight and adherent junctions within the epithelial layer to create a strong defense. Additionally, mucus production and transport, along with the secretion of antimicrobial peptides, work together to prevent pathogen invasion [4].

Molecules smaller than 11 Å exhibit a certain degree of permeability through the epithelial barrier via paracellular pathways, whereas larger (bio-)pharmaceuticals such as immunoglobulin G (IgG) have very limited permeability and rely predominantly on receptor-mediated transport mechanisms, such as transcellular IgG transport facilitated by the neonatal Fc receptor (FcRn). FcRn is essential in IgG transcytosis, recycling, and protection from lysosomal degradation. During transcytosis, IgG binds to FcRn, allowing transport in both apical-to-basolateral and basolateral-to-apical directions or recycling on the same side [5,6].

In addition to epithelial cells, the lamina propria, located beneath the epithelium, plays a significant role in IgG uptake through the airways. This layer contains blood and lymph vessels, glands, elastic fibers, and immune cells, which can bind immune complexes and monomeric IgG via their Fc fragments of IgG receptors (FCGR). This binding initiates various humoral effector functions, depending on receptor and cell type [7]. While immune cells exhibit the highest expression levels of FCGR, other tissues in the body also express these receptors [8]. For example, FCGRIIb facilitates IgG transcytosis through the placenta and possibly through the olfactory mucosa into the central nervous system. Further research is needed to elucidate the roles of other receptors in mucosal transport [9,10,11,12].

Developing robust and physiologically relevant in vitro models that accurately replicate the tracheal epithelium, including functional beating cilia, is critical for preclinical drug and formulation development. These models could enable a detailed understanding of the MCC of drugs and dosage forms, providing an invaluable platform for assessing the absorption, clearance, and, consequently, the availability, efficacy, and safety of (inhaled) drugs and their formulations at the mucosal airway surface. The ciliary activity and mucus production in these models will allow for more accurate predictions and clinical translations. Such advanced models are particularly relevant for respiratory drug development, where direct drug delivery to the airway mucosa has the potential to improve therapeutic outcomes and reduce systemic side effects.

Mucociliary clearance (MCC) is a vital defense mechanism of the respiratory tract, relying on the coordinated beating of motile cilia and the transport of mucus. It plays a crucial role in protecting against airborne pathogens and particulates, making its study particularly relevant for airway diseases such as chronic obstructive pulmonary disease (COPD), asthma, and cystic fibrosis, where MCC is often impaired [13,14]. In vitro models with beating cilia provide a valuable platform for understanding MCC, evaluating the impact of harmful exposures such as cigarette smoke, and supporting the development of new treatments [13,14]. Several MCC models have been developed using ciliated airway cells, with most relying on air–liquid interface (ALI) conditions to induce cell differentiation [15,16,17,18]. More complex systems, such as organoids [17,18,19] or tissue microengineering approaches [19,20], have also been explored. In vivo approaches predominantly focus on small animals like rats and mice [21,22]. Ex vivo approaches rely on tracheal tissue extracted from species like chickens [23] or mice [24]. Inoue et al. provide a comprehensive overview of publications that utilize tissues from various species [22]. In addition to the cell-based models, there are also various mathematics-based modeling approaches described, some of which are discussed by Smith et al. [25]. All these different approaches vary greatly in their choice of species, cell types, and methods of cultivation used. Depending on the research question, selecting a model that closely mimics in vivo conditions is essential for obtaining meaningful insights. Despite these advancements, there is no universally standardized method for MCC analysis, as each publication often describes a custom-tailored approach requiring specific hardware, software, or techniques [26,27,28,29,30,31]. For example, measuring CBF requires expensive high-speed cameras, which are not available or affordable for all laboratories [32].

Existing in vitro MCC models and assays, while innovative, are frequently limited in their transferability and standardization. This creates a pressing need for broadly applicable, robust, and cost-effective methods. Porcine (*Sus scrofa*) airway mucosa represents a promising alternative to human tissue due to its structural and cellular similarities to human airways and its availability in substantial quantities [33,34]. Additionally, porcine models offer a practical balance between physiological relevance and scalability. For example, porcine tracheal mucosa shares key features with the human respiratory immune system and has been shown to mediate immune interactions similar to those seen in humans [35]. Furthermore, human IgG has been shown to be transported into the porcine olfactory mucosa by porcine FcRn [35]. Additionally, Meurens et al. effectively highlighted the advantages and diverse applications of pigs in their review [36]. They described porcine models that played a valuable role in studying human infectious diseases due to their physiological similarities to humans, particularly in the skin, respiratory and digestive tracts, and immune systems. With advancements in porcine transgenesis, pigs are expected to become an increasingly accepted alternative to traditional mouse models for testing vaccines, immunostimulants, and disease mechanisms before advancing to primate studies or clinical trials [36]. Thus, porcine tissue and primary cells were used in this study.

Building on the protocol established by Fulcher and colleagues [15,37], we developed an advanced ciliated airway model derived from porcine tracheal mucosa. Cultured under ALI conditions to promote differentiation, this model features actively beating cilia and mucus production, enabling robust and reproducible MCC evaluation. By addressing the challenges of limited tissue availability, high costs, and the lack of standardized methodologies, this model provides a versatile tool for respiratory research, including drug development and toxicological assessments. The resulting model—particularly its cilia—was characterized and an in vitro MCC assay was developed. This study highlights the importance of in vitro models with beating cilia in respiratory drug-, formulation- and dosage form development.

## 2. Materials and Methods

### 2.1. Primary Cell Culture

For primary cell culture, two different models were used in this study. The first is the newly established cTMPC model, which contains beating ciliated cells. The second is the previously published TMPC DS model, which lacks beating cilia and serves together with the work from Fulcher et al. as the foundation for the newly developed cTMPC model [15,38]. Nevertheless, both models are derived from the same primary cells obtained from porcine trachea.

**Figure 1:**
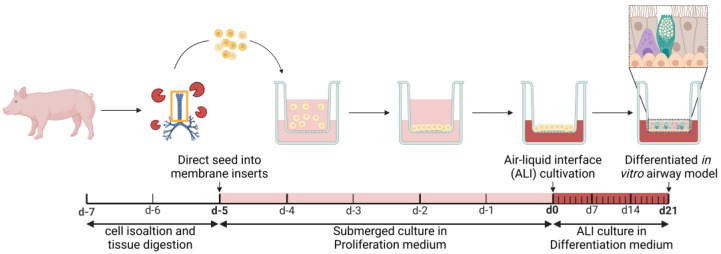
Workflow of cell cultivation procedures of ciliated trachea mucosal primary cells (cTMPC). For the extraction of cTMPC, all excess connective tissue was carefully removed and digested in Pronase. cTMPCs were then directly seeded into cell culture inserts and cultivated for 5 days under submerged conditions. After 5 days, the apical medium was removed, and cells were then cultivated for 21 days under air–liquid interface (ALI) conditions. See Supplementary Data S1 for details and Supplementary Table S1 for media composition. The sketch was created under license with BioRender.com.

For cTMPC cultivation, porcine tracheas from slaughtered 4–6-month-old pigs were obtained from a local butcher (Joas, Dietenheim, Germany) and further processed 1–2 h post mortem. Isolation and cultivation procedures were established based on previously published data with some modifications [15,16]. The detailed protocol for extraction and differentiation can be found in Supplementary Data S1. In brief, isolated cells were seeded at a density of 3 × 10^5^ cells/cm^2^ on the apical compartment of the porous inserts under submerged conditions. The composition of the proliferation medium corresponds to the Bronchial Epithelial Growth Medium (BEGM: see Supplementary Table S1) as previously described [37]. On day 5 of submerged culture, the cells were airlifted and received differentiation medium (see Supplementary Table S1 for media composition) via the basal compartment as previously described [37]. The cells were washed every 7 days to remove mucus. After 21 days of ALI cultivation, the cells were collected for characterization of permeability or MCC evaluation, unless otherwise specified. Figure 1 gives an overview of the extraction and cultivation of the cTMPC.

Unciliated trachea mucosal primary cells (TMPC DSs) were isolated and cultivated according to previously published data [38]. In brief, tracheal mucosa from 4–6-month-old pigs was collected and separated from the hyaline cartilage. The tissue samples were disinfected with Octenisept^®^ (Schülke & Mayr GmbH, Norderstedt, Germany), washed twice with prewarmed PBS, and transferred into T25 flasks containing 20 mL Pronase medium (1.4 mg/mL Pronase, Sigma-Aldrich, Taufkirchen, Germany). After incubation at 37 °C, 5% CO_2_, and 95% rH for 1 h, the supernatant containing TMPC was processed with debris removal solution (Miltenyi Biotec, Bergisch Gladbach, Germany) according to the manufacturer’s instructions. Following Pronase digestion and debris removal, cells were directly seeded into membrane inserts at a density of 10^5^ cells/insert using the same culture medium. As with the Flask method, cells were airlifted after 24 h and differentiated at the ALI for 21 days. All surfaces (flasks or membrane inserts) were pre-coated with 0.05 mg/mL rat tail collagen type I (Primacyte, Schwerin, Germany) at least 2 days before cultivation.

### 2.2. Histology

#### 2.2.1. Tissue Procession

For histological analysis, fresh porcine tracheal tissue was fixed in 10% (*v*/*v*) neutral buffered formalin (Carl Roth, Karlsruhe, Germany) overnight at 4 °C. TMPCs cultured on permeable inserts were fixed in 4% (*w*/*v*) paraformaldehyde (PFA) (Sigma-Aldrich, St. Louis, MO, USA) for 15 min at room temperature. Once fixed, the tissue was processed using the automatic tissue processor TP1020 (Leica Biosystems, Nußloch, Germany). In an ascending series of ethanol concentrations (trachea tissue: 70%, 80%, 95% ethanol, 1.5 h each and 3 × 100% ethanol, 1 h each; TMPCs cultured on porous inserts: 70%, 80%, 95%, 3 × 100% ethanol, 15 min each), the samples were dehydrated. Next, the tissue was processed in two xylene (Carl Roth, Karlsruhe, Germany) baths for a total of 3 h while cells grown on porous supports remained in the xylene baths for a total of 45 min. The tissue was then infiltrated with paraffin wax (Paraplast^®^, Leica Biosystems, Nußloch, Germany) at 56 °C for at least 4 h (cells grown on porous supports: 1 h). For the paraffin embedding, the embedding center HistoCore Arcadia (Leica Biosystems, Nußloch, Germany) was used. Using a rotary microtome (HistoCore Multicut, Leica Biosystems, Nußloch, Germany), the tissue block was cut into 5 µm sections and the sections were transferred on glass slides (Superfrost™ Plus Adhesion Microscope Slides, Epredia, Braunschweig, Germany). The slides were dried overnight at room temperature and then placed in an oven at 65 °C for 1 h to remove water and promote better tissue adhesion to the slides. Deparaffinization was achieved by repeated xylene incubations, each lasting 10 min. Rehydration was performed using decreasing concentrations of ethanol (2 × 100%, 95%, 90%, 80%, 70%) for 5 min each. The rehydration was completed by a final incubation in deionized water for 5 min.

#### 2.2.2. Alcian Blue Staining

Alcian blue staining to visualize acidic mucins was performed as previously described [39]. In brief, the slides were incubated in 3% (*v*/*v*) glacial acetic acid (Carl Roth, Karlsruhe, Germany) for 3 min and treated with 1% (*w*/*v*) Alcian blue 8 GX (Sigma-Aldrich, St. Louis, USA) in 3% (*v*/*v*) glacial acetic acid, pH 2.5 for 15 min. Excess dye was removed by washing the slides under running tap water for 5 min. Nuclear Fast Red (Sigma-Aldrich, St. Louis, USA) was used as a counterstain by incubating the slides for 2 min. After rinsing under running tap water, the sections were treated with increasing ethanol concentrations (95%, 2 × 100%, 2 min each) before subjecting them to xylene twice for 5 min each. Finally, the samples were mounted using Eukitt^®^ Quick-hardening mounting medium (Sigma-Aldrich, St. Louis, USA). Microscopical analysis was performed using a brightfield microscope Keyence BZ-X800 (Keyence, Osaka, Japan).

#### 2.2.3. Immunofluorescence (IF) Staining

After dewaxing and rehydration, the tissue sections were subjected to heat-induced epitope retrieval by immersion in sodium citrate buffer (10 mM sodium citrate (Sigma-Aldrich, St. Louis, MO, USA), 0.05% (*v*/*v*) Tween 20 (Carl Roth, Karlsruhe, Germany), pH 6) at 100 °C for 20 min. To prevent nonspecific binding, sections were blocked using 5% (*v*/*v*) donkey serum (PAN Biotech, Aidenbach, Germany) in IF buffer (0.25% (*v*/*v*) Tween 20 (Carl Roth, Karlsruhe, Germany), 0.2% (*w*/*v*) gelatin (Carl Roth, Karlsruhe) in PBS) for 60 min at room temperature. The incubation with primary antibodies involved an overnight incubation at 4 °C with the appropriate primary antibodies diluted in 2% (*v*/*v*) donkey serum in IF buffer. The following primary antibodies in the corresponding dilution were used: 1:100 rabbit-anti-ZO-1 (#NBP1-85047, Bio-Techne, Minneapolis, MN, USA), 1:1500 mouse-anti-acetylated α-tubulin (#T7451, Sigma-Aldrich, St. Louis, USA), 1:100 mouse-anti-TP63 (#sc-25268, Santa-Cruz Biotechnology, Dallas, USA), 1:400 mouse-anti-MUC5AC (#MA5-12178, Thermo Fisher Scientific, Waltham, MA, USA), 1:300 rabbit-anti-cytokeratin 5 (#NBP2-61931, Bio-Techne, Minneapolis, USA), 1:100 goat-anti-FOXJ1 (#AF3619, Bio-Techne, Minneapolis, MN, USA).

The following day, the sections were washed three times with PBS before incubating with the appropriate secondary antibodies diluted in 2% (*v*/*v*) donkey serum in IF buffer for 1 h at room temperature. The utilized secondary antibodies and the respective dilution were 1:300 donkey-anti-goat conjugated Rhodamine Red^TM^-X (Jackson ImmunoResearch Laboratories, Cambridgeshire, UK), 1:500 chicken-anti-rabbit conjugated Alexa Fluor^®^ 647 (Thermo Fisher Scientific, Waltham, MA, USA), 1:800 donkey-anti-mouse conjugated Alexa Fluor^®^ 488 (Jackson ImmunoResearch Laboratories, Cambridgeshire, UK). For counterstaining, 1 µg/mL 2-(4-Amidinophenyl)-1H-indole-6-carboxamidine (DAPI) (Thermo Fisher Scientific, Waltham, MA, USA) was added to the secondary antibody mixture to stain the nuclei. Next, the samples were washed five times with PBS.

To reduce autofluorescence, the sections were treated with a 10 mM copper(II) sulfate/50 mM ammonium chloride solution dissolved in deionized water for 10 min. Upon completion of staining, the sections were mounted onto microscope slides using Mowiol/DABCO embedding medium (33.33 g/L Glycerol (Carl Roth, Karlsruhe, Germany), 13.33 g/L Mowiol 4-88 (Carl Roth, Karlsruhe, Germany), 2.5% (*w*/*v*) 1,4-diazabicyclo[2.2.2]octane (DABCO) (Carl Roth, Karlsruhe, Germany) dissolved in 1:2 deionized water: TRIS-hydrochlorid, pH 8.5). The tissue sections were analyzed using the Keyence BZ-X800 fluorescence microscope.

The processing of primary cells for IF differed slightly from that of paraffin-embedded tissue sections described above. Specifically, the medium was removed from the basal compartment and the cell culture inserts were washed twice with PBS to eliminate cell debris, followed by fixation with either 4% (*w*/*v*) PFA or 1:1 acetone/methanol (Sigma-Aldrich, Waltham, MA, USA). All the subsequent staining steps were performed with the intact cell culture insert.

#### 2.2.4. Quantification of Ciliation, Cilia Length and Epithelial Thickness

To quantify the degree of ciliation on the apical epithelial surface, six randomly selected fields of view from each acetylated α-tubulin-stained insert were captured using a 20× objective (Keyence BZ-X800 fluorescence microscope, Keyance GmbH, Neu-Isenburg, Germany). The images were analyzed for cilia coverage using ImageJ/FIJI version 2.16 [40]. A fluorescence intensity threshold was applied, ensuring that only the ciliated regions were considered. The area above the threshold was measured for each image and expressed as a percentage of the total area. The cilia length and epithelial thickness were measured in Alcian blue-stained tissue sections at three fields of view, each with seven points (n = 21) using ImageJ/FIJI version 2.16 [40]. 

#### 2.2.5. Electron Microscopy

Transmission electron microscopy (TEM) and scanning electron microscopy (SEM) images of native tracheal tissue and cTMPCs were performed to gain deeper insights into the development of cilia and cell–cell connections.

SEM sample preparation and imaging were performed as previously described by Schütz et al. [41]. For this, the specimens were rinsed with PBS and fixed overnight at 4 °C in 2.5% (*v*/*v*) glutaraldehyde and 1% (*w*/*v*) saccharose in 0.1 M phosphate buffer (pH 7.3). The trachea was cut into small pieces and, similar to the cell culture inserts, post-fixed in 2% (*w*/*v*) osmium tetroxide solution at room temperature. Afterward, the transwell membranes were separated from the cell culture inserts and cut into smaller pieces. Following dehydration through a graded series of isopropanol, critical point drying was performed. The samples were then mounted onto aluminum SEM holders, and sputter-coated with 2 nm platinum. The specimens were analyzed using the S-5200 field emission scanning electron microscope (Hitachi, Tokyo, Japan) at 10 kV acceleration voltage.

TEM was performed as previously described by Olari et al. [42]. In brief, the tracheal mucosal tissue was removed from the underlying cartilage, or the cell culture inserts were fixed, post-fixed and dehydrated as described above for the SEM samples. Subsequently, the samples were block contrasted with uranyl acetate and embedded in Epon. Ultrathin (70 nm) sections were cut using an ultramicrotome Ultracut UC7 (Leica Biosystems, Nußloch, Germany). The sections were mounted on TEM grids and contrasted with lead citrate for 1 min. All specimens were analyzed using the JEM-1400 transmission electron microscope (Jeol, Tokyo, Japan).

### 2.3. Gene Expression Analysis Using RT-qPCR

To characterize the cell type composition in the cell model, a gene expression analysis of specific cell type markers was performed using real-time quantitative PCR (RT-qPCR).

For mRNA extraction from tissue and from cells grown on cell culture inserts, the RNeasy^®^ Plus Mini Kit (Qiagen, Hilden, Germany) was used. For tracheal tissue, 30 mg tissue samples were homogenized using a Potter–Elvehjem (Fisher Scientific, Waltham, MA, USA) homogenizer and additionally vortexed for 1 min. For cells cultured for 21 days under ALI conditions, 3–4 cell culture inserts of each condition were vortexed for 1 min and further processed according to the manufacturer’s instructions.

Contaminating genomic DNA (gDNA) was digested using the gDNA Removal Kit (Jena Biosciences, Jena, Germany) according to the manufacturer’s instructions.

Reverse transcription was performed using the LunaScript^®^ RT SuperMix Kit (New England Biolabs, Frankfurt am Main, Germany). The synthesis of coding DNA (cDNA) was performed using the SimpliAmp^®^ thermal cycler (Thermo Fisher, Waltham, USA) as per the manufacturer’s instructions. The generated cDNA was frozen at −20 °C until use.

If not stated otherwise, primer sequences in RT-qPCR analysis were used according to Martin et al. (see Supplementary Table S2) [38]. Additional primers for ciliogenesis and ciliated cells were designed using the primer designing tool “Primer-BLAST” by NCBI as described by Ye et al. [43]. Forward and reverse primers are listed in Table 1.

The RT-qPCR was performed using the Luna^®^ Universal qPCR Master Mix (New England Biolabs, Frankfurt am Main, Germany) following the manufacturer’s instructions. 10 ng of cDNA was used in a RT-qPCR reaction, and the respective primer pairs were added at a concentration of 0.25 µM each. The measurement was conducted in triplicates using the Roche Light Cycler^®^ 480 instrument (Roche, Basel, Switzerland).

The relative gene expression was quantified using the comparative (2^−∆∆Ct^) method described by Livak et al. [44]. The x-fold change was calculated according to Equations (1)–(4).

(1) $$\Delta c_{t_{sample}}=c_{t_{sample}}-c_{t_{GAPDH}}$$

(2) $$\Delta c_{t_{trachea\;tissue}}=c_{t_{trachea\;tissue}}-c_{t_{GAPDH}}$$

(3) $$\Delta \Delta c_{t}={\Delta c}_{t_{sample}}-{\Delta c}_{t_{trachea\;tissue}}$$

(4) $$x-fold\;change=2^{\left(-\Delta \Delta c_{t}\right)}$$

### 2.4. Transepithelial Electrical Resistance (TEER) Measurement

The TEER measurement was used to evaluate the barrier integrity of the cell layer during the differentiation phase in cell culture inserts using the EVOM3 TEER meter (World Precision Instruments, Sarasota, FL, USA) [45]. TEER solution (9 g/L sodium chloride, 183.78 mg/L calcium chloride and 2.388 g/L HEPES (all purchased from Carl Roth, Karlsruhe, Germany) dissolved in deionized water) was added at room temperature to both compartments (apical 350 µL and basal 500 µL). A cell culture insert without cells was utilized as baseline TEER. TEER values (Ω × cm^2^) were calculated by multiplying the measured resistance value (Ω) with the cell growth area of the 24 well cell culture inserts (0.336 cm^2^). A TEER value above 500 Ω × cm^2^ was considered an intact cell barrier.

### 2.5. Determination of Permeability

Permeability was assessed for the 4 kDa tracer fluorescein isothiocyanate-dextran (500 μg/mL FITC-dextran, Sigma-Aldrich, Germany) and a polyclonal intravenous immunoglobulin G (IgG) product (100 mg/mL; Panzyga^®^, Octapharma, Lachen, Switzerland). Prior to the permeation experiment, the TEER was determined and only cell culture inserts with a TEER ≥ 500 Ω × cm^2^ after 21 days ALI differentiation were used. The basolateral medium was replaced by 260 μL differentiation medium. A total of 100 μL of either FITC-dextran in PBS or IgG were added to the apical compartment. Next, 20 μL samples were taken after 1 h, 2 h, 4 h, 8 h, and 24 h from the basolateral side and replaced with 20 µL fresh differentiation medium to keep the basolateral volume constant. At the end of the permeability experiment, the TEER was measured again. FITC-dextran was quantified in a Tecan Infinite M200 Pro (Tecan Group Ltd., Männedorf, Switzerland) instrument at 490 nm/520 nm. IgG was quantified with an Fc-Fc sandwich ELISA using two polyclonal human Fc region-specific antibodies using a 1:7500 dilution of the capture antibody (Sigma-Aldrich, Darmstadt, Germany; #I2136) and a 1:50,000 dilution of the detection antibody (Sigma-Aldrich, Darmstadt, Germany; #A0170). The permeability coefficient (P_app_) was calculated according to Equation (5) with Δ[*c*]_b_ = basolateral concentration [μg/mL], [*c*]_a_ = apical concentration [μg/mL], Δ*t* = time of measurement [s], *A* = growth area of the membrane [cm^2^] and *V*_b_ = basolateral volume [mL].

(5) $$P_{app}\left(\frac{cm}{s}\right)=\frac{{\Delta [c]}_{b}\;*\;V_{b}}{A*\;{\left[c\right]}_{a}\;*\;\Delta t}$$

### 2.6. Mucociliary Clearance (MCC) Assay

For MCC analysis, porcine tracheal tissue and cTMPCs were used. cTMPCs were used only for MCC measurements on day 21 ALI culture with a TEER ≥ 500 Ω × cm^2^. To visualize the beating cilia, Lumogen-loaded mucoadhesive chitosan-coated poly(lactic-*co*-glycolic acid) (PLGA) nanoparticles with a mean diameter of 163.3 nm and a polydispersity index of 0.179 (MyBiotech, Überherrn, Germany, as previously described) were used [46].

**Figure 2:**
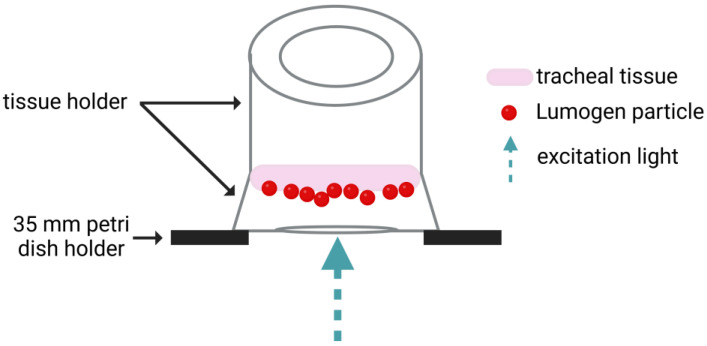
Graphical representation of the tissue holder used for mucociliary clearance analysis in tracheal tissue. Circular tracheal tissue sections were extracted from the whole trachea using a tissue puncher. The excised tissue was then positioned in the tissue holder with the apical surface oriented upwards. After securely closing the holder, a particle dilution was applied to the apical surface. Finally, the tissue holder was inverted and placed into the Keyence fluorescence microscope for analysis. The sketch was created under license with BioRender.com.

The particles were diluted 1:3000 in either PBS (100 mg/mL IgG) or 1% Hydroxypropylmethylcellulose (HPMC). Before the dilutions were applied on the tracheal tissue ~16 mm sections were punched out from the trachea using a tissue puncher (Vitrocell Systems, Waldkirch, Germany). After that, the tissue punches were incubated (37 °C, 5% CO_2_, and 95% rH) in DMEM: F12 (1:1) containing 20% FBS, 2 mM glutamine, 1% NEAA, 5 μg/mL amphotericin B, 200 μg/mL gentamycin sulfate and kanamycin, 20 U/mL penicillin, 20 μg/mL streptomycin, and 4.5 g/L Glu until use. The tracheal tissue was then placed into customized tissue holders (Vitrocell Systems, Waldkirch, Germany) and the corresponding dilutions were applied. Figure 2 gives an overview of the experimental set-up for the tracheal tissue. For cTMPCs, 20 µL of the suspension were added to the apical insert compartment of each ALI culture by pipetting. Movements of the fluorescent particles were recorded using the inverse Keyence BZ-X800 fluorescence microscope (Keyence, Osaka, Japan). The standardized recording settings with a 20× objective were 960 × 720 pixels with a resolution of 0.38 µm/pixel at a frame rate of 15 frames per second and a total duration of 30 s. Particle tracking was performed using the TrackMate plugin as described by Tinevez et al. in ImageJ/FIJI version 1.54 (National Institutes of Health, Bethesda, MD, USA, imagej.net) [47]. For each condition, three fields of view with visible particle movement were recorded from one cTMPC culture each. The following TrackMate settings were used: LoG detector, estimated object diameter: 1 µm, quality threshold: 10, Simple LAP tracker, linking max distance: 5 µm, gap-closing max distance: 5 µm, gap-closing max frame gap: 20. The median particle speed (µm/s) was calculated and compared between the three different solutions. Using this method, the influence of a highly concentration polyclonal antibody solution on MCC was determined.

Viscosity was measured using a HAAKE^TM^ Falling Sphere Viscometer C (#3560001, Thermo Fisher, Waltham, MA, USA) according to the manufacturer’s instructions.

### 2.7. Statistical Analysis

If not stated otherwise, results are presented as the mean ± standard deviation (SD). The data were analyzed for Gaussian normal distribution using a quantile–quantile plot and by performing the Anderson–Darling test. For a given Gaussian normal distribution, parametric tests were performed. For the comparison of two groups, a student’s *t*-test was performed. An ordinary one-way ANOVA with Tukey’s multiple comparison test was used to compare three or more groups. For non-parametric data, a Kruskal–Wallis test with Dunn’s multiple comparison test (three or more groups) or a Mann–Whitney test (two groups) was performed. Significance was determined by a p-value less than 0.05. The analyses were conducted using GraphPad Prism Version 10.1.0 (GraphPad Software Inc., La Jolla, CA, USA).

## 3. Results and Discussion

The aim of this study was to develop and characterize an in vitro airway epithelial model containing beating cilia and to evaluate its suitability for an MCC assay, thereby providing a robust platform for preclinical drug and formulation development. This advanced ciliated model (cTMPC) was additionally compared to a previously published model (TMPC DS) [38]. The previously published TMPC DS model lacks beating cilia and, therefore, does not allow for MCC analysis.

### 3.1. Determination of Epithelial Thickness and Cilial Length in the cTMPC Model

**Figure 3:**
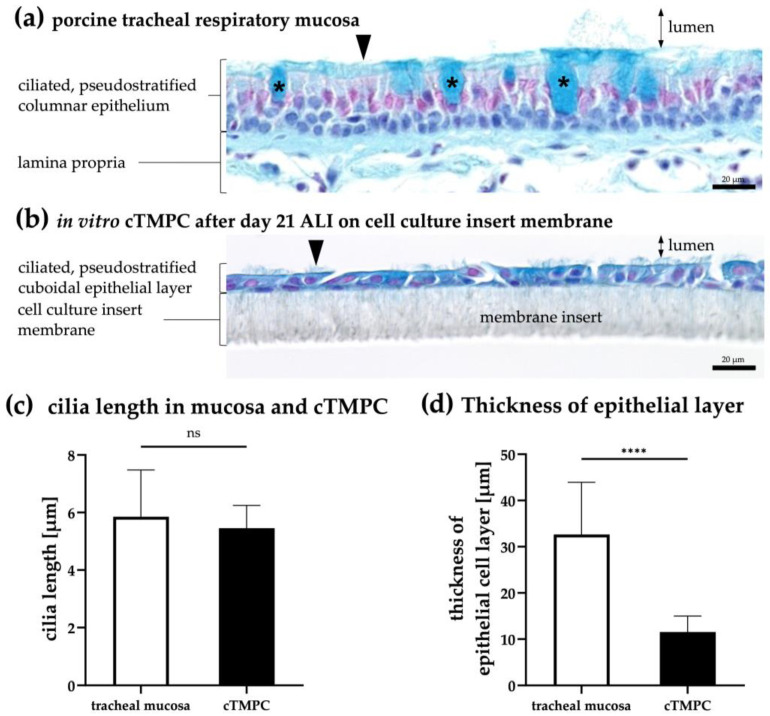
Histological evaluation of native porcine tracheal tissue and in vitro ciliated trachea mucosal primary cells (cTMPCs), quantification of cilia length and epithelial thickness. Alcian blue staining of histological cross sections (thickness: 5 μm) was assessed for respiratory mucosa from the trachea (**a**) and cTMPCs (**b**) cultivated for 21 days at ALI. (**a**) Native trachea tissue showed a typical ciliated pseudostratified columnar epithelium, with a basal cell layer underneath. Goblet cells are marked with an asterisk (*) and cilia with an arrow. Fibroblasts were observed in the lamina propria. In the cTMPC model (**b**) a ciliated pseudostratified cuboidal morphology of the apical cell layer was observed. Many but not all epithelial cells are ciliated in cTMPCs and no goblet cells were detected. Similarly to respiratory mucosa, the cTMPC models also display a thin basal cell layer underneath the epithelial layer. Representative images are shown. Scale bar: 20 μm. Cilia length (**c**) and thickness of the epithelial cell layer (**d**) were quantified at three different areas of independent samples (n = 7), while the cTMPCs originated from three different animals (N = 3). The cilia length of the in vitro cTMPCs is comparable to that of the native tracheal mucosal cilia (**c**). The epithelial thickness is significantly reduced in the cTMPC model. Values are presented as mean ± SD. ns: not significant, **** *p* < 0.0001 by ordinary one-way ANOVA.

Alcian blue staining was performed to compare the histological structure, cellular composition, epithelial thickness, and cilia presence and length of native tracheal tissue with cTMPCs cultivated for 21 days at ALI (Figure 3a,b). The cationic dye Alcian blue binds to negatively charged acidic polysaccharides such as acidic mucins found in goblet cells and on the epithelial surface [39]. Nuclear fast red was used for nuclei staining [48]. In addition, cilia length and the thickness of the epithelium were determined from the stained tissue and the cTMPCs on inserts (Figure 3c,d). To provide a clearer morphological comparison, fully differentiated cTMPCs (ALI day 21; Figure 3b) exhibited a pseudostratified cuboidal morphology, in contrast to the pseudostratified columnar morphology found in tracheal respiratory mucosa (Figure 3a). As the term “pseudostratified” implies, this epithelium appears multilayered but is formed from a single layer of cells. While all cells are anchored to the basement membrane, only some extend to the apical surface. The staggered arrangement of nuclei at varying heights creates the illusion of stratification, giving it a pseudostratified appearance [49]. This observation was also reflected in the quantified epithelial thickness (Figure 3d). The native tracheal epithelium exhibits a thickness of 32.6 ± 11.3 μm, which aligns well with previously reported values (30–50 μm) [50,51]. Notably, this thickness tends to decrease progressively from proximal to distal regions [52]. The observed differences in epithelial morphology further underscore the structural adaptation of the in vitro model compared to native tissue. In vitro cultivated cTMPC layers displayed only one-third of the epithelial layer in tissue (11.5 ± 3.5 μm) in concordance with previous reports [51]. Interestingly, at the edges of the cell culture insert, the cTMPC cells displayed a more polarized and columnar morphology; in total about 5% of all cells (Supplementary Figure S1).

When comparing cellular composition, distinct histological differences were evident between native and in vitro models. The histoarchitecture in cTMPCs was clearly reduced, and while, goblet cells and fibroblasts were clearly identified in the respiratory mucosa from trachea, no goblet cells nor fibroblasts were detected in cTMPCs. However, both the in vitro cTMPC epithelium model and the native tracheal epithelium consisted of two cell types: epithelial cells and basal cells underneath. By utilizing z-stack imaging of IF-stained basal cells, it was confirmed that basal cells were present in a deeper (abluminal) layer of the epithelium, while differentiated cells faced the luminal side and are connected by tight junctions (see Section 3.3 “Basal cell characterization and 3D structure of the cell model”).

A detailed examination of ciliation revealed structural differences between native and cultured epithelia. When examining the ciliation of the epithelial layer, it was evident from Figure 3a,b that the native tissue displayed a higher cilia density per cell compared to the in vitro primary culture. This difference in ciliation highlights the partial but incomplete differentiation of cTMPC cultures. Nevertheless, no significant difference could be identified between the length of native cilia from the trachea and those of cultured cTMPCs on inserts (Figure 3c); both have a similar average of 5 to 6 μm. The determined cilia lengths were within the range of 4–7 µm in accordance with those previously described for humans [29] and pigs [53].

### 3.2. Morphological Characterization of Barrier Integrity

**Figure 4:**
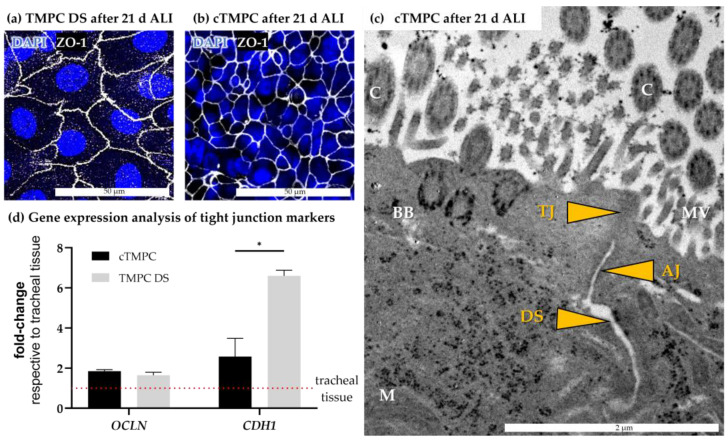
Evaluation of barrier integrity for trachea mucosal primary cells (TMPCs) cultured 21 days at air–liquid interface (ALI). Immunoreactivity against tight junction (ZO-1) in TMPC DS (**a**) and cTMPC (**b**) is shown in white. Nuclei were stained with DAPI and are shown in blue. Scale bar: 50 μm. Note the significantly smaller sizes of nuclei and cells in cTMPCs. Ultrastructural details of the junctional complex (**c**). The junctional complex in cTMPCs cultivated for 21 days at ALI cultivation was observed by TEM. Tight junctions (TJ), adherence junctions (AJ), and desmosomes (DS) were identified along the apicolateral borders of epithelial cells. Various other cell structures are also labeled: mitochondria (M), microvilli (MV), basal body (BB), and cilia. Additionally, the typical “9 + 2” axoneme arrangement in cilia from airway mucosa can be observed (C). Gene expression analysis of occludin (OCLN) and cadherin-1 (CDH1) (**d**). Both genes encode for proteins that are involved in barrier development. GAPDH was used as a housekeeping gene. X-fold change is shown as mean ± SD and was calculated in comparison to trachea tissue. The red dotted line indicates gene expression in porcine trachea tissue. cTMPCs: n = 2, N = 2. C: TMPC DS: n = 3, N = 3; * *p* ≤ 0.05, significant. If not mentioned otherwise in the graph, the *p*-value was >0.05, thus, no significant differences.

**Figure 5:**
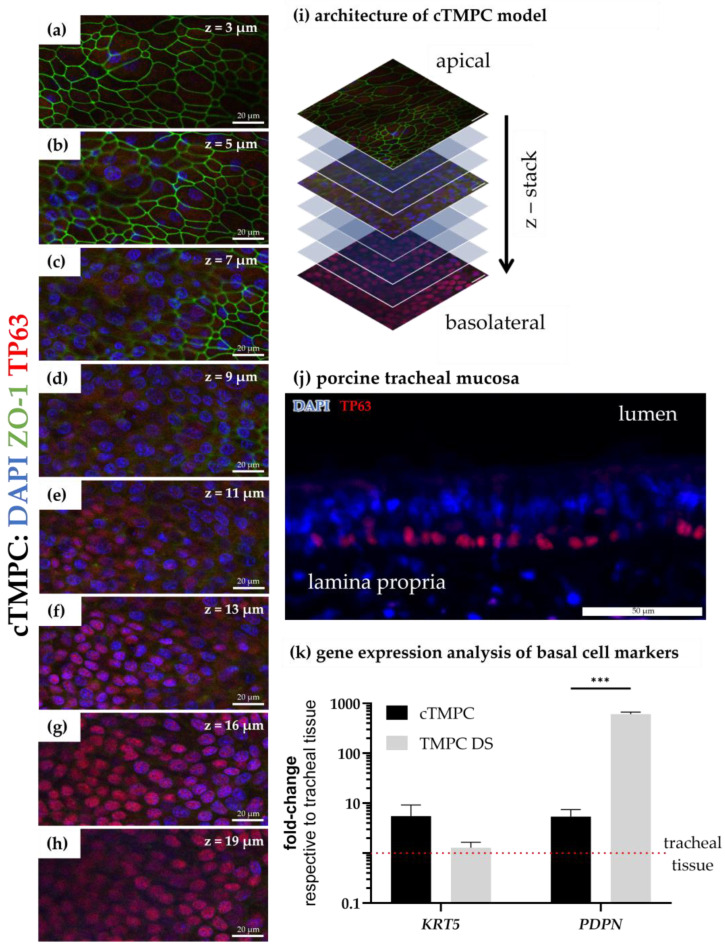
Characterization of basal cells in ciliated trachea mucosal primary cells (cTMPCs) cultured 21 days at air–liquid interface (ALI). Z-stack imaging series of the in vitro cultured cTMPCs to visualize tight junctions and basal cells (**a**–**h**). Z-stack images were captured at 2–3 μm intervals using confocal microscopy. Green represents zonula occludens-1 (ZO-1) immunoreactive tight junctions, red corresponds to TP63 immunoreactive basal cells, and blue corresponds to nuclear DNA DAPI stain. Tight junctions are present at the apicolateral side between cells while basal cells are located at the basolateral side. Scale bar: 20 μm. Overview of z-stack images and orientation of the apical and basolateral side (**i**). Immunoreactivity of ZO-1 and TP63 in respiratory mucosa from pig; scale bar 50 µm (**j**). Gene expression analysis of *KRT5* and *PDPN* (**k**). Both genes encode for proteins that are mainly expressed in basal cells. GAPDH was used as a housekeeping gene. X-fold change is shown as mean ± SD and was calculated in comparison to tracheal tissue. The red dotted line indicates gene expression in porcine trachea tissue. *** *p* ≤ 0.001, significant; if not mentioned otherwise in the graph *p*-value was > 0.05 and therefore there was no significant difference; cTMPC: n = 2, N = 2. C: TMPC DS: n = 3, N = 3.

In epithelial tissues, the neighboring epithelial cells are tightly connected to each other, forming a critical physical barrier. This tight epithelial barrier acts as a vital first-line defense mechanism protecting the organism from pathogen invasion and limiting drug permeation [54,55,56]. To assess junctional integrity and barrier formation in TMPC DS (Figure 4a) and cTMPCs (Figure 4b) following 21 days of ALI culture, immunofluorescence (IF) analysis confirmed the presence of the tight junction-specific protein ZO-1. Both, cTMPC and TMPC DS displayed a cobblestone-like appearance. The number of cells exhibiting this morphology increased during ALI cultivation, forming a continuous band of ZO-1 immunoreactivity without interruptions. Furthermore, z-stack imaging confirmed that ZO-1 was localized at the sub-apical regions of cell-to-cell borders, demonstrating the expected position of tight junctions within both epithelial barrier models (Figure 5). Interestingly, the ZO-1 staining revealed significant differences in nuclear and cellular size between TMPC DSs and cTMPCs (Figure 4a,b). TMPC DSs exhibited a significantly larger nuclear diameter (18.48 ± 3.41 µm) compared to cTMPCs (8.38 ± 2.02 µm; **** *p* ≤ 0.001; Student’s *t*-test). This difference can be attributed to various factors, including the developmental stage, state of cell differentiation, external influences, and cellular transformation [57]. As a result, TMPC DSs may exhibit less differentiation than cTMPCs, resulting in a larger nucleus due to the loosely packed chromatin, which facilitates extensive mRNA transcription.

TEM provided insights into the location of junctional complexes maintaining the paracellular barrier of epithelia and consisting of the following intercellular contacts listed from apical to basal direction: tight junctions (TJs), adherens junctions (AJs), and desmosomes (DS) [58]. Figure 4c shows the ultrastructural details of cTMPCs cultivated for 21 days at ALI and the above-mentioned components of the junctional complexes could be clearly identified along the apicolateral border. In addition to junctional complexes, other cellular structures were clearly visualized including mitochondria (M), microvilli (MV), basal bodies (BB) anchoring cilia; and cilia (C) involved in mucociliary clearance and sensing environmental stimuli. This detailed ultrastructural analysis highlights the organized architecture and functionality of cTMPCs under ALI culture conditions, emphasizing the role of tight junctions, adherence junctions, and desmosomes in maintaining epithelial barrier integrity.

The results obtained from ZO-1 immunoreactivity and TEM were verified by expression analysis of genes involved in tight junction formation including occludin (*OCLN*) and cadherin-1 (*CDH1*) for both cell culture methods (Figure 4d). RT-qPCR revealed a 1.6- to 6.6-fold higher expression of both genes compared to the tracheal mucosa, from which the cell models were derived. While *OCLN* was comparable in both models, TMPC DS revealed a significantly higher expression of *CDH1* in comparison to cTMPC. The higher expression of tight junction genes in both cell models is likely a direct consequence of their reduced cellular composition, which consists predominantly of epithelial cells forming tight junctions. In contrast, the tracheal respiratory mucosa, with its diverse cell types in the epithelial layer and lamina propria, exhibits a comparatively lower expression of these genes due to the reduced proportion of cells actively forming tight junctions.

Transepithelial electrical resistance (TEER) is a standard method for evaluating the barrier function in cell culture models of epithelial barriers. For the previously published TMPC DS model, only cultures on membrane inserts that developed a TEER ≥ 500 Ω × cm^2^ after 21 days at ALI conditions were used for subsequent experiments [38]. Thus, the TEER of cTMPCs was evaluated and compared to the TMPC DS model. After 21 days ALI cultivation, both cTMPCs (822.19 ± 206.91 Ω × cm^2^) and TMPC DSs (779.95 ± 314.91 Ω × cm^2^) displayed a TEER above the 500 Ω × cm^2^ threshold and are in the range of 700 to 1200 Ω × cm^2^ described for human tracheal and bronchial cells [59,60] as well as for primary swine tracheal epithelial cells (~800 Ω × cm^2^) [61]. Statistical analysis revealed no significant difference between the two models. It is estimated that TEER correlates to the integrity of the tight junctions [62].

In summary, both cell models showed proper barrier development on gene and protein levels and proper organization of junctional complexes. Interestingly, z-stack analysis of cTMPC cultures revealed that similar to the architecture of respiratory mucosa ZO-1, immunoreactivity was observed only in the apical third (Figure 5a–i). This demonstrates that the cTMPC model differentiated successfully under ALI conditions into a 3D model of the respiratory epithelium.

### 3.3. Basal Cell Characterization and 3D Structure of the Cell Modell

Basal cells are the multipotent progenitor cells of the airway epithelia and reside along the basement membrane [63]. The cells are instrumental in maintaining epithelial homeostasis and are involved in repair and regeneration following injury [64]. In culture, basal cells survive due to their inherent stem-like properties, including robust proliferative capacity and adaptability to in vitro conditions. Under appropriate culture conditions, basal cells are also in vitro progenitors prior to differentiation into various epithelial cell types, such as ciliated cells and goblet cells [65]. To characterize the 3D architecture and cellular composition of the cTMPC model, basal cells were identified using their specific markers: tumor protein p63 (TP63, by immunoreactivity), podoplanin (*PDPN*), and keratin 5 (*KRT5*, both by RT-qPCR).

Immunoreactivity against TP63 was investigated in z-stacks of cTMPCs after 21 days under ALI conditions (Figure 5a–i). At the bottom of the cell culture and attached to the membrane insert, a continuous cell layer of TP63-positive cells was found (Figure 5e–i). The architecture of TP63-positive cells within the epithelial model corresponds to that observed in the respiratory mucosa (Figure 5k) and as described in both native airway epithelia and differentiated airway epithelial cells from various animal species [15,16,66,67,68,69]. The basal cell layer in the differentiated cTMPCs suggests that this 3D model may also possess the potential for tissue repair and regeneration [70].

Z-stack analysis demonstrates clearly the spatial organization into two sublayers within the cTMPC 3D model with an apical ZO-1 immunoreactive layer (Figure 5a–d) and a TP63 immunoreactive layer (Figure 5e–h) underneath [71]. Wu et al. described ZO-1 to be located along the apicolateral surface of rabbit ciliary epithelium [72]. In tracheal mucosa, basal cells were also located close to the basal lamina underneath an apical cell layer (Figure 5j) as previously described by Evans et al. [73].

The gene expression analysis revealed a higher expression for both basal cell markers in both, the TMPC DS and cTMPC models compared to the tracheal tissue (Figure 5k). This could be due to the fact that during the cell extraction, mainly basal cells survive, proliferate and serve as progenitors for differentiated cells [74]. Therefore, there is a higher ratio of basal cell mRNA in total isolated mRNA in the cell models than in the tissue, resulting in a lower Δc_p_ value. While there was no significant difference in *KRT5* expression between TMPC DS and cTMPC, TMPC DS had a significantly increased *PDPN* expression. PDPN is also a tumor marker likely to be involved in incomplete differentiation [75]. Analogously to the increased nuclear size, this might be an indication that the cells of the TMPC DS model may not be fully differentiated.

### 3.4. Mucus Production and Secretion

The analysis of mucus is of critical importance as impaired mucin composition can alter the physicochemical properties of mucus, which in turn affects MCC, and therefore, drug delivery. Mucus also acts as a physical barrier to most pathogens [28]. Mucus consists of water, proteins, salts, lipids, cell debris and most importantly mucins, further subdivided into cell-associated mucins and secreted mucins. One of the most prominent secreted, gel-forming mucins of the airways is MUC5AC, which is mainly secreted by goblet cells [54,76,77,78].

**Figure 6:**
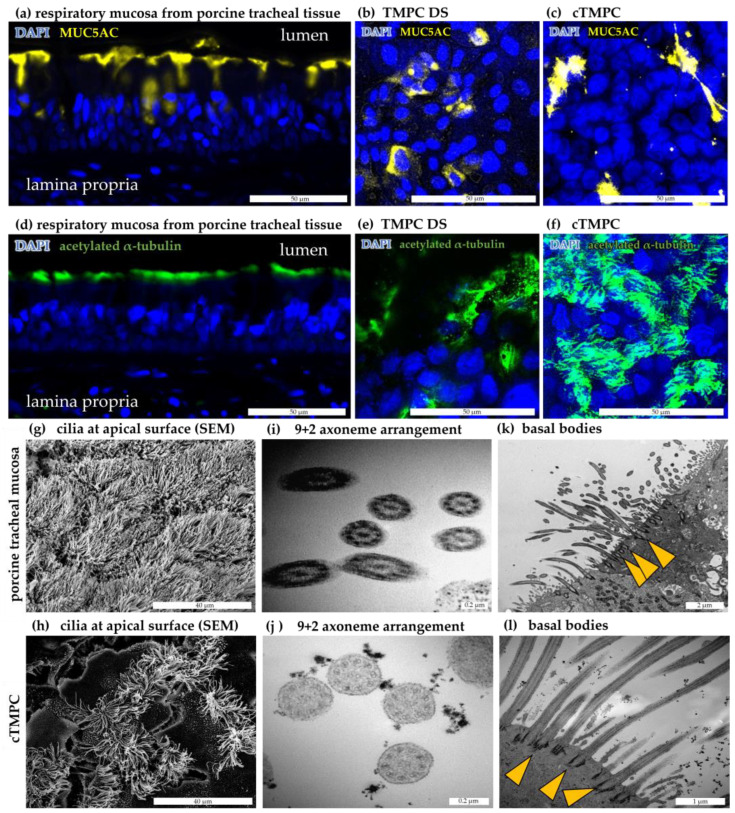
Characterization of MUC5AC (yellow) production in porcine trachea (**a**), (TMPC DS (**b**) and cTMPC (**c**); both 21 d ALI). Immunoreactivity against acetylated tubulin (green) as marker for cilia in porcine trachea and (**d**), (TMPC DS (**e**) and cTMPC (**f**); both 21 d ALI). Nuclei were counterstained with DAPI in blue. Scale bars: 50 µm (**a**–**f**). While both models show a reduced mucin production compared to respiratory mucosa, cTMPCs have a significantly increased number of ciliated cells. Ultrastructural analysis of the cilia by scanning electron microscopy (SEM) in porcine airway mucosa (**g**) and cTMPC after 21 ALI (**h**) demonstrate the ciliated cells in the cell model, though significantly lower than in mucosa. Transverse sections analyzed by transmission electron microscopy (TEM) show the typical “9 + 2” axoneme arrangement in cilia from airway mucosa (**i**) and cTMPC (**j**). Longitudinal sections analyzed by TEM show basal bodies anchoring the cilia in the cell highlighted by orange arrowheads in mucosa (**k**) and cTMPC (**l**).

Immunoreactivity against MUC5AC was observed in respiratory mucosa from porcine trachea as a network of secretions coating the apical epithelial surface, ducts of glands and with less intensity also within cells (Figure 6a), similar to what has been previously reported [69,78,79]. A robust immunoreactivity against MUC5AC likely to be located in secretory vesicles was found in the TMPC DS model after 21 d ALI (Figure 6b). While typical polarized goblet-shaped cells could not be identified in cTMPC cultures (Figure 3b), a similar pattern as in TMPC DS was observed in cTMPCs, but a lower number of cells were positively stained (Figure 6c). The presence of mucus has previously been described in in vitro differentiated airway epithelial cell models derived from pigs [6,51,61,80,81], humans [74,78,79,82], or other animal species [16,67,69]. However, compared to the tracheal tissue, both models displayed a lower number of MUC5AC-producing cells with cTMPCs displaying the lowest number (Figure 6a–c). Earlier studies described the formation of goblet cells in a relatively late phase of ALI cultivation. For instance, Prytherch et al. described that goblet cells in human epithelial cells started to form after ALI day 15, although mucus was visible earlier [74]. Schagen et al. reported the identification of goblet cells from week 3 at ALI, but they became much more evident after 5–7 weeks [83]. Thus, pro-longed ALI cultivation should be considered to induce goblet cell differentiation and possibly thereby increase mucin production.

Both cytoplasmic and extracellular mucins were visualized by MUC5AC staining, but it should be noted, that due to the numerous washing steps of the staining procedure, it could not be assured that all extracellular secreted mucus was fully fixed to the cellular surface and thereby preserved to be fully detectable. Consequently, these images represent a qualitative evaluation rather than a quantitative one.

Therefore, different mucins were analyzed by RT-qPCR (Supplementary Table S3). As expected from the lower number of MUC5AC-positive by IF in both cell models, gene analysis also revealed a 300- to 750-fold decrease in expression of *MUC5B* in both models and a decrease in *MUC5AC* in cTMPC compared to tracheal mucosa (TMPC DS: 0.0037 ± 0.0019; cTMPC: 0.0013 ± 0.0097). Specifically, *MUC5AC* was only 4-fold decreased in TMPC DS, but over 40-fold decreased in cTMPC confirming the IF data (TMPC DS: 0.225 ± 0.1458; cTMPC: 0.022 ± 0.0042). A similar *MUC5AC* expression pattern was described previously at RNA transcript level [84] and at protein expression level [16,84,85]. Enhanced mucus production in cTMPC could be achieved by optimizing the culture medium. Previously, it was demonstrated that an increase in the EGF concentration [86] and an elevation of retinoic acid concentration [85,87,88,89,90] enhance mucus production. Additionally, reducing hydrocortisone concentration [91] and the absence of triiodothyronine [89,91,92] led to increased mucus production. Moreover, the cytokine interleukin 13 (IL-13) can induce the goblet cell formation [93,94,95].

Not only mucins were reduced; club cells are considered to contribute to the formation of airway surface liquid, as they are described to produce watery secretions that are necessary for the formation of the periciliary layer [28]. Typical club cell-specific markers are *SCGB1A1* and *SCG3A2*, both encode for members of the secretoglobulin family, and their expression was analyzed. In parallel to the expression of mucins, club cell markers were clearly reduced in both models with a 20-fold reduction in TMPC DS and a 10,000-fold reduction in cTMPC for *SCGB1A1* (TMPC DS: 0. 0522 ± 0.0738; cTMPC: 0.0001 ± 0.0002) (Supplementary Table S3). For *SCGB3A2*, a 12- and 17-fold reduction was observed (TMPC DS: 0. 08 ± 0.109; cTMPC: 0.0582 ± 0.0434). No significant differences in expression were detected between cTMPC and TMPC DS (TMPC DS: n = 3, N = 3; cTMPC: n = 2, N = 2). Club cells are mainly found in the distal airways such as the bronchioles [96,97,98].

Interestingly, cTMPCs showed a higher mucus signal after 7 days ALI, which decreased over time until 21 days ALI. This observation correlated with the *MUC5AC* gene expression. In cTMPCs, MUC5AC accumulation was observed to decrease over time while ciliation increased. Jong et al. and You et al. also found that there was a negative correlation between cilia development and the development of secretory cells [16,85]. A possible cause for this phenomenon could be attributed to the Notch pathway. In recent years, this pathway has come into focus and is considered an important pathway that affects progenitor cell fate and differentiation events in the airway epithelium [99]. The activation of Notch facilitates the differentiation of basal cells into secretory cell types, such as goblet cells, while its inhibition is required for the differentiation of ciliated cells [100,101]. However, to confirm the activity of the Notch pathway during TMPC differentiation, experimental evidence is needed. The Notch receptor is a cell surface receptor activated by binding to its ligands Delta or Jagged on neighboring cells [102]. Ligand binding leads to the cleavage and release of the notch intracellular domain (NICD) [102]. NICD can be detected, for example, by immunoblotting, as it has a lower molecular weight than the intact Notch receptor. After enzymatic cleavage (e.g., γ-secretase), NICD then travels to the nucleus to regulate transcriptional complexes [102]. Upon activation of the Notch pathway, the *HEY1* gene is upregulated. *HEY1* is a transcription factor that leads to the repression of the cell gene expression program and the acquisition of a secretory cell precursor identity [99,103]. Further downstream genes that play a role as transcription factors in the differentiation of goblet cells include, e.g., *SPDEF* [99]. Conversely, when cells do not receive an active Notch signaling trigger, other genes such as *MCIDAS* and further downstream, *TP73* and *FOXJ1*, are activated, leading to their differentiation into ciliated cells [99,103]. These recent discoveries show many possibilities to investigate cell fate developments in TMPC in future experiments.

### 3.5. Formation and Ultrastructure of Cilia in cTMPC

The motivation to improve the previously published TMPC DS model was the low formation of cilia, which were not beating since the beating ciliated cells represent the key component of MCC responsible for removing pathogens and inhaled particles [28]. Therefore, the cultivation technique of Fulcher and co-workers was adapted for tracheal primary cells with the cTMPC model as the result. To visualize cilia formation, immunoreactivity against the cilia component acetylated α-tubulin was performed [104]. A longitudinal section of porcine tracheal mucosa shows as control a robust immunoreactivity at the apical side (Figure 6d). Representative images of ciliated cells after 21 days of ALI culture show the typical hairy-like structure (Figure 6e,f). It should be noted that ciliated cells were present, but a rather rare event in TMPC DS. When quantifying the percentage of ciliated area per area epithelium the number of ciliated cells was ~1% in TMPC DS while ~60% of the surface in cTMPC was ciliated, similar to what has been reported for bovine [67] and ovine [69] airway epithelial cells after 21 day ALI. In vivo, roughly 70% of the porcine tracheal surface is covered with cilia [105]. Thus, the cTMPC in vitro model mimics well the in vivo situation.

Reasons for reduced cilia generation and differentiation in TMPC DS could be that the concentration of mitogenic substances in the TMPC DS cultivation medium might be comparatively high. Mitogens stimulate cells to enter the cell cycle (mitosis) and therefore proliferation by overcoming intracellular checkpoint mechanisms that block cell cycle progression [106]. However, cell differentiation is usually triggered by proliferation arrest and exit from the G0 phase and stimulate cell cycle progression, preventing differentiation into, e.g., ciliated cells [106]. A low concentration of mitogens is therefore beneficial for cell differentiation [106]. Hormones or growth factors such as EGF have a mitogenic potential and can influence ciliogenesis [107]. These substances might be lower in cTMPC medium (e.g., 0.5 ng/mL EGF and 10 μg/mL BPE) compared to the TMPC DS medium (unknown EGF concentration, as FBS was used).

Interestingly, prior studies have indicated that continuous overstimulation of cells with mitogenic signals did not lead to excessive proliferation but rather, it triggered the activation of cell cycle checkpoint mechanisms that induce either cell-cycle arrest or programmed cell death (apoptosis) to prevent the development of tumor cells [106]. For this reason, it is not surprising that instead of a multilayered epithelium, a cuboidal partly squamous unciliated epithelium formed in TMPC DS. Other studies were also able to identify the formation of squamous undifferentiated epithelia at a higher EGF concentration (25 ng/mL) [87,88,108]. Optimizing the TMPC DS medium may be necessary to induce ciliogenesis by replacing FBS containing medium with a defined medium, like for cTMPC. For example, reducing bovine pituitary extract or EGF [15,87,109], or increasing retinoic acid [67,87,89,110,111] but also the application of the Notch inhibitor DAPT [112] can enhance ciliogenesis.

Therefore, TMPC DS was not considered for further electron microscopy analysis and later MCC assessment. Complementary to IF, cilial structures were further characterized by SEM and TEM for respiratory mucosa and cTMPC. Surface analysis using SEM revealed a strong ciliation in the respiratory mucosa from the porcine trachea (Figure 6g). As expected from IF, the density and uniformity of ciliation were generally less pronounced in cTMPC (Figure 6h) compared to tracheal mucosa, similar to observations from previous studies [82]. In porcine tracheal mucosa, SEM revealed a more or less complete cilia coverage of the luminal epithelial surface. Gan et al. reported complete cilia coverage in large airways of minipigs [113]; in humans, a cilia coverage of 85–90% was also observed [114,115]. However, other publications state a lower value of approximately 45% ciliated cells [29,59,96]. The difference may be attributed to the authors examining the total epithelial tissue composition rather than the luminal cilia coverage of the epithelium. In general, the use of different methods for cilia quantification complicates direct data comparison. Notably, previous studies also indicate that the number of ciliated cells remained constant until day 33 [74] or even until ALI day 42 [67]. Nevertheless, the morphology of the cilia was mostly similar to the mucosa. However, an altered shape of the cilia’s distal tip was observed in some ciliated cells in cTMPC cultures. Further, cTMPC showed a thin twisted distal tip. It is possible that the altered morphology is due to the axoneme still assembling. Reynolds et al. have shown that during ciliogenesis, the central microtubule singlets are capped by a so-called flagellar tip complex that extends farther than the neighboring microtubule doublets and the other axonemal microtubule assembles thereafter [116]. Alternatively, it could be a ciliopathy in which the distal tip does not assemble properly, perhaps due to a disturbed intraflagellar transport system, but this leaves much room for speculation.

Ultrastructural TEM analysis demonstrated that the “9 + 2” arrangement of axonemes in cilia from freshly isolated mucosa (Figure 6i) and in cTMPC (Figure 6j) is conserved in almost all forms of eucaryotic motile cilia. This arrangement consists of nine microtubule doublets surrounding two central microtubule singlets, known as the central apparatus [28]. Ciliary motility is achieved by the regulated action of the outer and inner axonemal dynein arms associated with the doublet microtubules. Periodic contact with the neighboring doublets causes them to slide relative to each other. This sliding is restricted by protein bridges between neighboring doublets and by the basal anchoring of the axoneme, which leads to a bending movement [117]. The adequate position of basal bodies anchoring the cilia was also revealed by TEM in both tracheal mucosa (Figure 6k) and cTMPC (Figure 6l), similar to other ALI airway epithelial cell cultures [67,69,74,111].

A significantly enhanced cilia formation was observed in cTMPC cultures compared to TMPC DS. Therefore, the expression of genes involved in ciliogenesis and cilia structure was analyzed (Supplementary Table S4). In summary, only minor differences, if any, were observed in most analyzed transcripts and the expression levels in cTMPC were similar to those of tracheal mucosa. Expression of cilia structure genes (molecular ruler (*CCDC40*), radial spoke (*RSPH4A*), inner and outer dynein arms (*DNAH5* and *DNALI1*) and genes associated with ciliogenesis (*MCIDAS*, *DEUP1*, *TP73*) were unaltered to mildly decreased in both models compared to mucosa. The only exception was the transcription factor FOXJ1 involved in ciliogenesis. *FOXJ1* was upregulated in cTMPC over 1000-fold while being below the detection limit in TMPC DS. Bukowy-Bieryłło et al. conducted a similar study on ALI-cultivated human nasal epithelial cells, using the commercial differentiation medium PneumaCult™ [84]. They observed an earlier increased expression of *FOXJ1*, *RSPH4A*, and *CCDC40* as early as ALI day 3, while the gene expression of *DNAH5* and *DNALI1* started to rise from day 6 onwards. This highlights the importance of *FOXJ1* in cilia formation. Further, it strengthens the assumption of *FOXJ1* being the master regulator of motile ciliogenesis [118]. In addition, as suggested by other studies [84,119], media composition and cell species origin, among other factors, can influence the gene expression profile and timing during ciliogenesis, hence making a direct comparison of the two in vitro models challenging.

Analysed together, the results gathered here demonstrate that cTMPC shows a high and uniformly distributed cilia coverage on the cell culture inserts by ALI day 21. Moreover, it was also shown that the cilia are structurally integer and motile. Conversely, TMPC DS showed strongly impaired ciliation, despite showing comparable gene expression pattern for cilia related genes, except *FOXJ1*.

### 3.6. Assesment of Ciliary Clearance in cTMPC Compared to Specimens from Airway Mucosa

**Figure 7:**
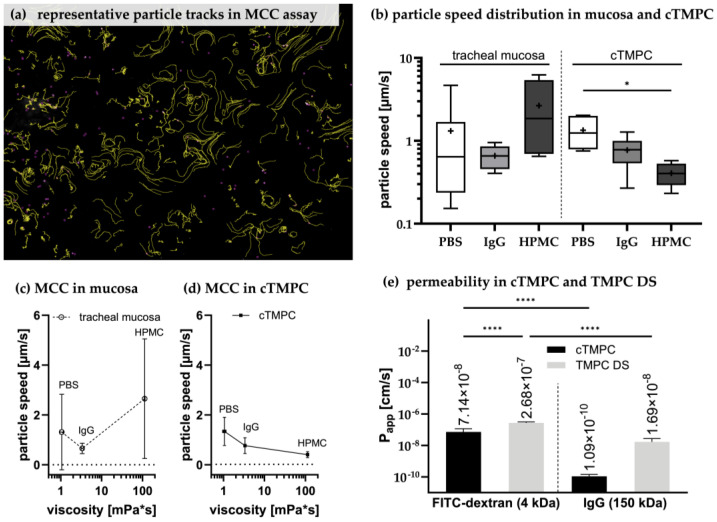
Using primary models for the preclinical evaluation of drugs, formulation, and dosage forms. Representative tracks of the chitosan-coated lumogen-capsulated PGLA particles (PBS on cTMPC) (**a**). Each yellow line represents a track of a single particle (violet dots). Particles were tracked using ImageJ plugin version 1.54 [47,128] and the median particle speed was determined per sample (**b**). The individual median is shown as a line in a box plot with min. to max. whiskers; the mean is displayed as ‘+’. While in the mucosa, median and mean differ largely, both more and less overlay in cTMPC (tracheal mucosa: PBS: n = 14, N = 3; 100 mg/mL IgG: n = 17, N = 3. cTMPC: PBS: n = 10, N = 3; 1% HPMC: n = 7, N = 3; 100 mg/mL IgG: n = 11, N = 3. Statistical analysis was performed using a nonparametric unpaired Mann–Whitney test for tracheal mucosa and a nonparametric unpaired Kruskal–Wallis test for cTMPC. * *p* ≤ 0.05; if not stated otherwise, there was no significant difference). Due to large variances, no dependence of particle speed with viscosity was observed for MCC in mucosa; mean ± SD (**c**). By contrast, in MCC on cTMPCs, a significant impact of viscosity on particle speed (* *p* ≤ 0.05; two-way ANOVA) was observed (**d**). Characterization of the barrier integrity of ciliated trachea mucosal primary cells (cTMPCs) and TMPC DS. (**a**) P_app_ was determined based on the permeability with 500 µg/mL FITC-dextran or 100 mg/mL IgG (**e**). Only cell culture inserts with a TEER ≥ 500 Ω × cm^2^ were used. (cTMPC FITC-dextran n = 60–66, N = 3; cTMPC IgG n = 60–62, N = 3. TMPC DS FITC-dextran n = 15–25, N = 3; cTMPC IgG n = 3–5, N = 1; **** *p* ≤ 0.0001).

MCC is a critical function and key component of the airway epithelium’s barrier mechanism and highly relevant for the clearance of mucosal drugs and their dosage forms [28]. After successful evaluation of mucin production and cilia formation in cTMPCs, an assay was developed to assess the clearance by beating cilia in cTMPCs compared to excised respiratory mucosa from the trachea with a short post mortem delay. The TMPC DS model was not considered here, as insufficient numbers of inactive cilia were detected. Chitosan-coated particles are known for their mucoadhesive properties [120], allowing for the assessment of mucus propulsion facilitated by cilia. Fluorescent and mucoadhesive (Chitosan-coated lumogen-labeled poly(lactic-co-glycolic) acid; PLGA) nanoparticles were placed onto the apical surface of in vitro cTMPC (ALI day 21) or ex vivo specimens from tracheal mucosa. The propelled particles were tracked using fluorescence microscopy video imaging. To validate this assay, formulations with different viscosities were used. The particles were either spiked into a hydrogel (1% hydroxypropylmethylcellulose; HPMC), a viscous solution of highly concentrated antibodies (100 mg/mL IgG) or PBS as a reference. Figure 7a shows the tracks (yellow lines) of several nanoparticles (small violet dots) propelled by beating cilia. A representative video illustrating particle movement is available as Supplementary Video S1. Based on these tracks, the analysis tool TrackMate determines the median speed for each particle and thereby the median particle speed was calculated for each of the three formulations investigated. In total, 900–23,000 tracks were recorded for each tested sample (cTMPC or mucosa with one of the three formulations), ranging from fast-moving particles up to particles that hardly move at all. For biostatistical analysis, the median particle speed from all tracks of each sample (in vitro cTMPC or ex vivo mucosa) was used for further analysis.

The ex vivo mucosa model exhibited high variability, making it less suitable for reliable MCC analysis compared to the more consistent in vitro cTMPC model (Figure 7b). For example, particles applied in PBS on ex vivo mucosa displayed a mean particle speed of 1.32 ± 1.517 µm/s (median: 0.6413 µm/s; n = 14 determinations), while in a solution of 100 mg/mL IgG, the mean speed decreased to 0.66 ± 0.208 µm/s (median: 0.657 µm/s; n = 17). However, the use of the hydrogel HPMC revealed an interesting artifact in the ex vivo model, with particle speed showing high heterogeneity (mean: 2.65 ± 2.403 µm/s; median: 1.860 µm/s; n = 8). This effect was not observed in the cTMPC model.

The challenges encountered with the ex vivo tissue stemmed from its more complex experimental setup compared to the cTMPC analysis. Unlike the cTMPC model, where the cell culture inserts are much thinner and can be easily placed on a slide, ex vivo samples require positioning within a customized tissue holder. This additional step, combined with the need for inverse imaging microscopy, further complicated the analysis process. Additionally, in the ex vivo setup, the emitted light had to pass through the thicker tissue before reaching the detector, further reducing image clarity and complicating video recordings. In contrast, the thinner cell culture inserts used in the cTMPC model facilitated easier and more effective video capture. Moreover, the application of hydrogel was hindered by its high viscosity, which prevented the formation of a uniform thin layer on the thicker and uneven surface of the ex vivo tracheal tissue. These factors collectively increased the error rate and variability of the ex vivo MCC model. As a result, no significant differences in particle speed were observed due to the high variability of the data (Figure 7b). Furthermore, a clear correlation between particle speed and viscosity was not evident, primarily because of the inconsistent results with HPMC (Figure 7c).

In contrast, the cTMPC model enabled more robust and reproducible MCC analysis with significantly lower variability. As expected, higher viscosity formulations resulted in reduced particle speeds in this model, illustrating a clear inverse relationship between viscosity and MCC efficiency (Figure 7d). Particles in PBS (mean: 1.34 ± 0.568 µm/s; median: 1.240 µm/s; n = 6) moved faster than those in viscous IgG solution (mean: 0.77 ± 0.316 µm/s; median: 0.781 µm/s; n = 8) or HPMC hydrogel (mean: 0.41 ± 0.121 µm/s; median: 0.404 µm/s; n = 7). An overall effect of viscosity on particle speed was observed (* *p* ≤ 0.05; Figure 7d), with the difference between PBS and HPMC being significant. However, the reduction in speed for IgG relative to PBS was not statistically significant, suggesting that the sensitivity of the MCC assay remains limited when distinguishing between viscosities close to 1.05 mPa·s (PBS) [121] and 3.35 mPa·s (100 mg/mL IgG) [122]. Nevertheless, a comparable correlation between increasing viscosity and inhibition of MCC was reported previously [55,123,124].

The in vitro MCC assay using cTMPCs provides a reliable platform for testing drug formulations and assessing their effects on MCC. While improvements to the tissue-based ex vivo method may be feasible, our efforts prioritized the cTMPC model due to its superior performance and reproducibility. Nonetheless, the particle speeds observed in our experiments were lower than those reported in other studies. For instance, Robinot et al. reported particle speeds of 8.9 µm/s [125], and Roth et al. described speeds of 11.6 µm/s [115]. Even higher velocities, around 90 µm/s, have been documented for MCC in human native trachea [126]. These discrepancies likely reflect differences in experimental setups, such as the use of larger, non-mucoadhesive beads (e.g., 1 µm [115] or 30 µm [125]), or rigorous cell washing to minimize excess mucus before bead application. This aligns with the findings of Henning et al., who demonstrated that material properties and the associated particle surface chemistry can influence MCC [127].

Despite these differences, the cTMPC model demonstrated a strong inverse relationship between viscosity and particle speed, underscoring its potential for evaluating inhalable drugs and formulations. It is essential to consider factors such as molecular weight, chemical structure, and molecular charge, as these properties can significantly influence MCC efficiency. This highlights the importance of careful formulation design to optimize drug delivery via the mucociliary system.

### 3.7. Permeability of cTMPC Compared to TMPC DS to Evaluate Drug Transport

Permeability and drug uptake across epithelial airway models are relevant for preclinical screening. Therefore, FITC-dextran with a mean molecular weight of 4 kDa [129] and polyclonal IgG with a mean molecular weight of 150 kDa [130] were used as a fluorescent tracer to determine the apparent permeability coefficient (P_app_) giving a hint of the flux through a barrier [131] (Figure 7e). The P_app_ depends on the molecular weight of the molecule and the cell line/cell type used [132,133,134,135]. FITC-dextran was shown to permeate predominantly via intercellular junctions [136], whereas intracellular transport of IgG via Fc receptors was described [11]. The determined P_app_ of both molecules was lower in cTMPCs compared to TMPC DSs (**** *p* ≤ 0.0001 for FITC-dextran). In summary, cTMPCs revealed P_app_ values of 7.14 × 10^−8^ ± 4.29 × 10^−8^ cm/s for FITC-dextran and 1.09 × 10^−10^ ± 0.342 × 10^−10^ cm/s for IgG while TMPC DS displayed 2.68 × 10^−7^ ± 0.421 × 10^−7^ cm/s for FITC-dextran and a comparable P_app_ of 1.69 × 10^−8^ ± 1.05 × 10^−8^ cm/s for IgG (Figure 7e). For molecules with a molecular size of ~4 kDa, previous studies using Calu-3 cells demonstrated P_app_ of ~10^−7^ cm/s [137], suiting well the P_app_ obtained for FITC-dextran in this study. For Calu-3, Sibinovska et al. divided the permeability in high (~>0.75 × 10^−6^ cm/s), moderate (~0.75 × 10^−6^ cm/s > x >0.25 × 10^−6^ cm/s), and low (~<0.25 × 10^−6^ cm/s) [132]. According to this classification, cTMPCs revealed low permeability for both molecules. TMPC DS got moderate permeability for FITC-dextran and low permeability for IgG. The lower P_app_ of cTMPCs may result from the beating cilia impeding the molecule transport and possible receptor binding. The data obtained for FITC-dextran after 24 h in both models can be compared to previously published data for olfactory and respiratory porcine primary cells [6]. Ladel et al. stated amounts of 0.55–2.85 µg FITC-dextran after 24 h in their primary airway cell models [6], comparable with data from this study.

Interestingly, this difference in P_app_ of FITC-dextran between cTMPCs and TMPC DSs is not reflected by the similar TEER of both models, which they displayed after 21d ALI culture. However, 24 h later at the end of the permeability assessment, the TEER of cTMPC was somewhat lower, but still above the threshold (592.91 ± 116.46 Ω × cm^2^). By contrast, the TEER of TMPC DS was significantly lower and beneath the threshold after 24 h exposure to FITC-dextran or IgG (302.76 ± 165.99 Ω × cm^2^). Thus, the barrier of cTMPC remains intact for 24 h, reducing the potential for paracellular transport. For TMPC DS, the barrier integrity was significantly reduced, thus, the probability of paracellular transport was increased which may explain the observed differences in FITC-dextran P_app_ (Figure 7e). A drop in TEER during a permeation experiment was previously reported in a canine kidney model [138]. Further, we observed that the medium during permeation should only be changed to a different one (like minimal essential media) if the cultivation medium interferes with subsequent analytics. This limits the risk of a TEER drop.

As Fc receptors are described to mediate IgG uptake, gene expression analysis of different Fc receptors was performed for both primary models to investigate the observed differences of IgG P_app_. FcRn was reported to be expressed in various epithelial cells, including bronchial epithelium, alveolar epithelium of the lungs, nasal mucosa, and olfactory epithelium [11,139,140,141,142]. Both cell culture models showed a comparable expression of *FCGRT*, the gene encoding FcRn, when compared to native tracheal mucosa (Supplementary Table S5). Therefore, the slight reduction in IgG transport observed in cTMPC is unlikely to be due to the number of FcRn receptors. FCGR (Fc-gamma receptors) have also been speculated to be involved in IgG transport and trafficking. *FCGRIA* is found on the surface of monocytes, dendritic cells, macrophages, or activated neutrophils [143]. *FCGRIIA* and *FCGRIIB* are expressed by macrophages, neutrophils, eosinophils, and DC [144], and last but not least, *FCGRIIIA* is found on the surface of natural killer cells and macrophages [145]. Many of these immune cells are non-adherent and are likely to not survive under ALI conditions [146]. Additionally, the medium used for primary cell cultivation is not designed to support the growth of immune cells. Nevertheless, FCGRs are also described to be expressed in epithelial cells [10,147], in primary airway models [38], and keratinocytes, astrocytes, liver cells, sensory neurons, endothelial cells, and salivary gland epithelial cells [8]. As the TMPC cell models lack lamina propria and the immune cell-containing lymphoid follicles, it was not surprising that the expression levels of all *FCGR* reduced compared to respiratory mucosa from the trachea. Thus, *FCGRIA* levels were ~10-fold reduced in cTMPC and ~40-fold in TMPC DS. *FCGRIIIA* levels were reduced by over 100-fold in cTMPC and over 1500-fold in TMPC DS. Interestingly, levels of *FCGRII,* which was reported to contribute to IgG trafficking, were ~10–50-fold reduced in TMPC DS compared to mucosa, while not detectable in the cTMPC model. Further studies are needed to unravel if the expression levels of the *FCGR* family are the cause for the low transport in cTMPC with 0.4 ± 0.12 µg IgG (n = 60–62, N = 3) detected from the basolateral compartment after 24 h.

A comparable study in porcine primary airway cells from the olfactory mucosa with 500 µg/mL IgG1 detected ~3.75 µg to be transported to the basolateral compartment after 24 h [11]. Even though, in the present study, a 200-fold dose was applied, the results only show a ~13-fold increase in TMPC DS (48 ± 27.8 µg IgG within 24 h; n = 3–5, N = 1), although the expression levels of all Fc receptors are similar in the olfactory primary cells (unpublished data) than in TMPC DS. Thus, it is likely that this high dose resulted in saturation of all Fc receptors with IgG and thereby to a ceiling effect in IgG transport. Nevertheless, the different binding affinities of IgG subtypes (IgG1, IgG2, IgG3, and IgG4) to the different Fc receptors affecting overall IgG transport should also be considered [148].

Table 2 presents a concise summary of the key findings for cTMPCs, comparing them with previously published data on human airway models. Additionally, information on CBF has been included, as this parameter is commonly used to describe cilia movement.

## 4. Conclusions

A sufficient number of motile cilia was observed exclusively in cTMPCs, while non-motile cilia were present only in negligible amounts in TMPC DS. Consequently, a robust MCC assay was developed using cTMPCs, demonstrating higher sensitivity and improved reproducibility compared to assays performed with mucosa specimens.

Further validation of this MCC assay is necessary, along with a comprehensive analysis of the potential influence of cilia on permeability and transport studies. These efforts will help establish a reliable platform for respiratory research and the development of inhalable drug formulations.

Key parameters of the cTMPC model, such as cilia length, epithelial thickness, TEER, tight junction integrity, mucus production, presence of beating cilia, particle speed in the MCC assay, and Papp, were thoroughly evaluated. Additionally, RT-qPCR was performed for various specific markers. These well-defined characteristics serve as valuable reference points for future studies, further reinforcing the model’s reliability and applicability in respiratory research.

## Figures and Tables

**Table 1:** Primer sequences used for gene expression analysis in the porcine airway mucosa. Sequences for genes involved in ciliogenesis and sequences for ciliated cells are shown. F: forward, R: reverse, bp: base pairs.

Target	Gene	Sequence (5′-3′)		Product Length (bp)
Genes involved in ciliogenesis	*DEUP1*	F	GCAGCGCAGTGCAGGTTAAA	75
		R	TGGGCTTGGTTCTCCATGTC	
	*MCIDAS*	F	CTGGACAGGAAGTTCGCTCC	170
		R	CCGAGTAACGAGGAGCAGTC	
	*TP73*	F	TCAGGCGGCACCAGAGTG	134
		R	AAAATAGGTGCTGTCCGGCT	
Ciliated cells	*DNALI1*	F	CCAAGCTCCCCTCAACTTCC	96
		R	TGTCCTCCACCCATTCCCTTG	
	*DNAH5*	F	TGCTCTGACTGGGGTTTGTG	91
		R	GTTGAAGCTCACCTCCCGAT	
	*RSPH4A*	F	CCAGGGAAATTTAGAAGGAGCTG	120
		R	TCCGTGCCCAAACCAACTCCAG	
	*CCDC40*	F	TGGAGAAAAAGCGCATCCTGCA	125
		R	GTCCATGGACTTGGCTTGATGC	
		R	GCATAGTCCGAAAGGGGAGG	

**Table 2:** Summary of key findings in ciliated tracheal mucosal primary cells (cTMPC) and comparison with human in vitro airway models. This table presents a comparison of cTMPC with human in vitro airway models, highlighting key characteristics such as cilia length, epithelial thickness, transepithelial electrical resistance (TEER), tight junction integrity, mucus production, beating cilia, particle speed in the mucociliary clearance (MCC) assay, cilia beating frequency (CBF), and apparent permeability coefficient (P_app_).

Characteristic	cTMPC	Human In Vitro Airway Model
cilia length [µm]	5–6	4–6; [149] *
epithelial thickness [µm]	11.5 ± 3.5	35–40; [149] *
TEER [Ω×cm^2^]	822.19 ± 206.91	700–1200; [59] *
tight-junctions	yes	yes; [149] *
mucus production	yes	yes; [149] *
beating cilia	yes	Yes; [149] *
particle speed MCC [µm/s]	0.41–1.34	~ 40–180; [30] *
CBF [beats/s]	not measured	~ 6–18; [30,149] *
P_app_ [cm/s]–4 kDa	7.14 × 10^−8^ ± 4.29 × 10^−8^	2.20 × 10^−7^ ± 0.30 × 10^−7^; [150] **^/#^
P_app_ [cm/s]–150 kDa	1.09 × 10^−10^ ± 0.342 × 10^−10^	0.36 × 10^−7^ ± 0.22 × 10^−7^; [150] **

* data for human bronchial epithelial cells. ** data for human alveolar epithelial cells. ^#^ data for 4.4 kDa molecule.

## Data Availability

The original contributions presented in this study are included in the article/Supplementary Material. Further inquiries can be directed to the corresponding author.

## Supplementary Materials

The following supporting information can be downloaded at: https://www.mdpi.com/article/10.3390/pharmaceutics17040462/s1, Data S1: Isolation, Cultivation and Differentiation of cTMPC; Table S1: Media composition; Table S2: RT-qPCR primer sequences of different markers for the porcine airway mucosa; Figure S1: Histological evaluation of cTMPC cultures; Table S3: Gene expression analysis of goblet and club cell related genes; Table S4: Gene expression analysis of cilia related genes; Video S1: Representative video for the evaluation and tracking of the chitosan-coated lumogen paticles on cTMPC; Table S5: Gene expression analysis of Fc-receptors.
