# Supplementary material for: Development and Characterization of a Primary Ciliated Porcine Airway Model for the Evaluation of In Vitro Mucociliary Clearance and Mucosal Drug Delivery
Source: Pharmaceutics. 2025 Apr 2;17(4):462. doi: 10.3390/pharmaceutics17040462 (PMC12030231; doi:10.3390/pharmaceutics17040462)
Supplement: Supplementary file 1 [file pharmaceutics-17-00462-s001.zip › pharmaceutics-3501931-supplementary.pdf]

Supplementary Tables and Data

Supplementary Data S1: Isolation, Cultivation and Differentiation of cTMPC:

Using scissors and tweezers, all excess connective tissue was carefully removed. The tracheas were then washed by placing them in conical tubes filled with sterile phosphate-buffered saline (PBS; Carl Roth, Karlsruhe, Germany) and gently inverting them several times. Subsequently, the tracheas were transferred to fresh conical tubes containing 30 ml of digestion medium (Minimal Essential Medium (MEM; Biowest, Nuaillé, France), 15 mM HEPES (Carl Roth, Karlsruhe, Germany), 100 U/ml Penicillin, 100 µg/ml Streptomycin (Thermo Fisher, Waltham, MS, USA), and freshly added 1.4 mg/ml Pronase® protease from *Streptomyces griseus* (Merck Millipore, Burlington, MS, USA). The tracheas were incubated at 4 °C in the conical tube, kept upright.

After 48 h, pre-warmed fetal bovine serum (FBS, Capricorn Scientific, Ebsdorfergrund, Germany) was added to a final concentration of 10% (v/v) to the digestion medium to inactivate the Pronase® enzymatic activity and inverted gently ten times. Next, the trachea was transferred to fresh MEM medium supplemented with 10% (v/v) FBS and inverted again ten times. This procedure was repeated for a third iteration. The medium containing the detached epithelial cells was pooled and centrifuged at 500 x g for 5 min. Then, the supernatant was discarded, and the cell pellet was washed with sterile PBS and centrifuged again at 500 x g for 5 min.

In the next step, the epithelial cells were directly seeded on collagen type IV-coated porous cell culture inserts. ThinCert® porous cell culture inserts with a pore size of 0.4 µm and a pore density of 1 x 10^8 pores/cm^2 (Greiner Bio-One, Kremsmünster, Austria) were used. 0.05 mg/ml collagen type IV solution (Thermo Fisher, Waltham, USA) was applied apically to the cell culture inserts and incubated for 2 h at 37 °C or overnight at room temperature. The coated inserts were also stored long-term at 4 °C by sealing the plates with Parafilm® (VWR International GmbH, Darmstadt, Germany).

Dissociated cells, isolated as described above, were seeded at a density of 3 x 10^5 cells/cm^2 in 150 µl of proliferation medium on the apical compartment of the porous inserts. 400 µl of proliferation medium was added to the basal compartment. The preparation of the proliferation medium corresponds to the composition of the Bronchial Epithelial Growth Medium (BEGM) previously described by Fulcher *et al.* [23]. For the detailed medium composition, see Supplementary Table S1.

2 days after cell seeding, the medium was exchanged. The old medium was removed, the cell layer was washed with 200 µl Hank's Balanced Salt Solution (HBSS) without Ca^{2+} and Mg^{2+} (Capricorn Scientific, Ebsdorfergrund, Germany), and fresh proliferation medium was added to the apical (150 µl) and basal (400 µl) compartments to maintain submerged cultivation. On day 5 of submerged culture, the airlift was performed. For this purpose, the proliferation medium in both the apical and basal compartments was removed, the cells were washed with 200 µl of HBSS. Afterwards, 400 µl of differentiation medium was added to the basal compartment. The preparation of the differentiation medium corresponds to the composition of the ALI medium as previously described by Fulcher et al. [37]. For details see Supplementary Table S1.

Every 2 - 3 days, the basal differentiation medium was exchanged. The cells were washed with 200 µl of HBSS every 7 days to remove accumulated mucus. After 21 days of air-liquid interface (ALI) cultivation, the cells were collected for analysis, unless otherwise specified.

Supplementary Table S1: Media composition based on Fulcher et al., 2005 [15]. The final concentration of the additives in media is listed. Components marked with an asterisk (*) were supplemented additionally and are not mentioned in the original protocol by Fulcher et al., 2005.

	Medium/additive	Final concentration in media	Supplier, cat. number
	Basic medium	Proliferation medium: LHC Basal<br>Differentiation medium: 1:1 LHC Basal: Dulbecco's Modified Eagle Medium (DMEM), high glucose	LHC Basal: Thermo Fisher Scientific, #12677019<br>DMEM-H: Biowest, #P0103
	Bovine serum albumin (BSA)	0.5 mg/ml	Carl Roth, #8076
	Insulin	0.87 μM	Capricorn Scientific, #INS-K
	Transferrin	0.125 μM	Thermo Fisher Scientific, #11107018
	Bovine pituitary extract (BPE)	10 μg/ml	Thermo Fisher Scientific, #13028014
	Epidermal growth factor (EGF)	Proliferation medium: 25 ng/ml<br>Differentiation medium: 0.5 ng/ml	Peprotech, #AF-100-15
	Retinoic acid	0.5 μM	Sigma-Aldrich, #R2625
	Isoprenaline*	Proliferation medium: 0.3 μM<br>Differentiation medium: not contained	Sigma-Aldrich, #I5627
	Penicillin<br>Streptomycin	100 U/ml<br>100 μg/ml	Thermo Fisher Scientific, #15140122
	Primocin™*	Proliferation medium: 100 μg/ml<br>Differentiation medium: not contained	InvivoGen, #ant-pm
	Hydrocortisone	0.21 μM	Thermo Fisher Scientific, #A16292
	Triiodothyronine	0.01 μM	Sigma-Aldrich, #T2877
	Epinephrine	2.7 μM	Sigma-Aldrich, #E4250
	Phosphorylethanolamine	0.5 μM	Thermo Fisher Scientific, #367420050
	Ethanolamine	0.5 μM	Thermo Fisher Scientific, #451762500
	Zinc sulfate	3.0 μM	Sigma-Aldrich, #Z0251
Stock 4	Ferrous sulfate	0.0015 mM	Sigma-Aldrich, #F8048
Stock 4	Magnesium chloride	0.6 mM	J.T Baker, #2444
Stock 4	Calcium chloride	0.11 mM	Sigma-Aldrich, #C3881
Trace elements	Selenium	30 nM	Sigma-Aldrich, #S5261
Trace elements	Manganese	1 nM	Sigma-Aldrich, #M5005
Trace elements	Silicone	500 nM	Sigma-Aldrich, #S5904
Trace elements	Molybdenum	1 nM	Sigma-Aldrich, #M1019
Trace elements	Vanadium	5 nM	Sigma-Aldrich, #398128
Trace elements	Nickel sulfate	1 nM	Sigma-Aldrich, #N4882
Trace elements	Tin	0.5 nM	Sigma-Aldrich, #S9262

Supplementary Table S2: RT-qPCR primer sequences of different markers for the porcine airway mucosa. Martin et al. 2024 [38].

Marker for	mRNA target	Forward Primer (5′-3′)	Reverse Primer (5′-3′)
**House-keeping**	*GAPDH*	GTTGTGGATCTGACCTGCCG	CAGCCCCAGCATCAAAGGTAG
**Tight junctions**	*OCLN*	TTAAAAACGTGTCGGCAGGC	GCATAGTCCGAAAGGGGAGG
	CDH1	TTCAAGAAGCTGGCGGACAT	AGTCCCCTAGTCGTCCTCAC
**Ciliated cells**	*FOXJ1*	CTTCCAGAACCTTCCTTTGGC	TCGAATGTGGTGTCTGGCTCA
	TUBB4B	CCGCTCTTCGGCTTTTCTTAC	CGCTGATCACCTCCCAAAACTTG
	CETN2	TCGAATGTGGTGTCTGGCTCA	AGTGCCCTCATTGCCACCTTA
**Airway epithelial cells**	*MUC1*	AAGATCCCACCACCAGCTAC	GCTAAGGTTTGATGCAAGGGG
	MUC4	TCACACCACCACTTCAGTCC	TGTCATAGTGTTTCCACCCAGG
**Fibroblasts**	*COL3A1*	CCGAGCTTCCCAGAACATCA	CCCCAGTGTGTTTAGTGCAAC
	LUM	GGCTCCTTTGATGGACTGGT	GCCAGAAGGCAGTTTGGTCA
**Basal cells**	*PDPN*	ACTGTAGGAAGCACAACGCA	CCGTCCCCAAGCCATCTTT
	KRT5	GGCGAGGAGTGCAGATTGAG	GGTGTTTGTGACGACCGAGA
**Goblet cells**	*MUC5AC*	CTGACCAAGAGCTCCGTCCT	CGCAGGTCTTGTTGGCGTAT
	MUC5B	CACCCACATCGTCCTCAAGA	TGTCCTCTCCGTTCCACACA
**Club cells**	*SCGB1A1*	GACAGTGTGTTAAAACTCCAGGAAA	TCAGGGCCTCCAGAGATGAA
	SCGB3A2	GGCATTTCTGTTGAGCACCTG	CAAGTATGAGAGAGCCTCCAGC
**Fc-receptors**	*FCGRT*	GAGAGTGACATCGGCCCCAA	CCAACCACTGGCATGGAGGA
	FCGR1A	CATGGGAAGCAAGACCCTGA	CTGCGCTTGATGACCTTTCC
	FCGR2A	GCAAAGTTGAGGAGTTGGGGG	TGAACCCAGTGAGATTGGGCA
	FCGR2B	GGAGCCCATCTTGCTGCGGT	AGCTGGACCCTTGGACAGTG
	FCGR3A	GCTGGCACACACGCTGAAGA	CCCTGGCACTTCAGAGTCACA

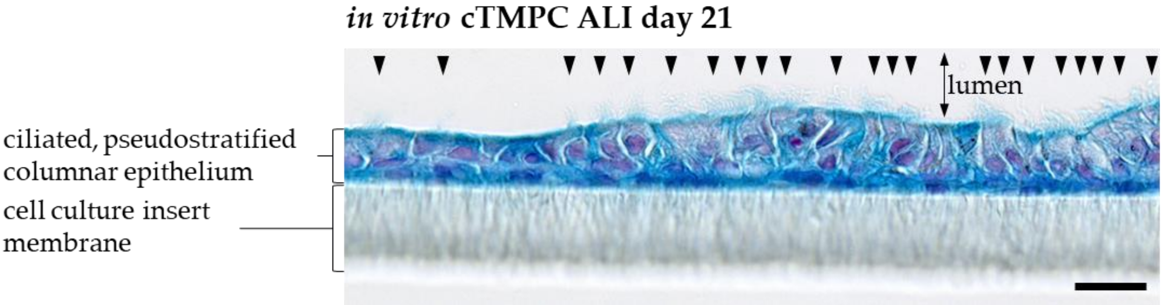

Supplementary Figure S1: Histological evaluation of ciliated trachea mucosal primary cell (cTMPC) cultures at the edges of the cell culture insert visualized by Alcian blue staining of paraffin-embedded tissue cross-sections. At the edges of the insert, the cTMPC cells displayed a more polarized and columnar morphology. In total, this was observed for about 5% of all cells. Alcian blue/Nuclear Fast Red staining of in vitro cTMPC cultured 21 days at air-liquid interface (ALI) on porous membranes is shown. Alcian Blue stains the acidic mucins found in goblet cells and on the epithelial surface. The airway lumen and the cell culture insert membrane are labeled. Cilia are marked with an arrowhead. Shown is a representative image. Scale bar: 20 µm.

Supplementary Table S3: Gene expression analysis of goblet and club cell related genes (*MUC5B, MUC5AC, SCGB1A1, SCGB3A2*). *GAPDH* was used as housekeeping gene. cTMPC: n = 2, N = 2. C: TMPC DS: n = 3, N = 3. x-fold change is shown as mean ± SD and was calculated in comparison to tracheal mucosa.

mRNA target	cTMPC	TMPC DS
MUC5B	0.013 ± 0.010	0.004 ± 0.002
MUC5AC	0.022 ± 0.004	0.225 ± 0.146
SCGB1A1	0.0001 ± 0.0002	0.052 ± 0.074
SCGB3A2	0.058 ± 0.043	0.080 ± 0.109

Supplementary Table S4: Gene expression analysis of cilia related genes (*FOXJ1, MCIDAS, DEUP1, TP73, TUBB4B, CETN2, DNALI1, DNAH5, RSPH4A, CCDC40*). *GAPDH* was used as housekeeping gene. cTMPC: n = 2, N = 2. C: TMPC DS: n = 3, N = 3. x-fold change is shown as mean ± SD and was calculated in comparison to tracheal mucosa.

**mRNA target**	**cTMPC**	**TMPC DS**
FOXJ1	1641.86 ± 571.09	below detection
MCIDAS	0.445 ± 0.170	0.571 ± 0.266
DEUP1	0.390 ± 0.090	0.468 ± 0.093
TP73	0.212 ± 0.106	0.254 ± 0.135
TUBB4B	0.920 ± 0.189	0.093 ± 0.178
CETN2	0.638 ± 0.069	0.135 ± 0.121
DNALI1	0.924 ± 0.075	0.954 ± 0.143
DNAH5	0.451 ± 0.199	0.543 ± 0.384
RSPH4A	0.702 ± 0.011	0.547 ± 0.466
CCDC40	0.989 ± 0.261	1.128 ± 0.754

Supplementary Video S1 Representative video for the evaluation and tracking of the chitosan-coated lumogen paticles on ciliated tracheal mucosa primary cells (cTMPC). Fluorescent paticles (red) were spiked 1:3000 into 1% hydroxypropylmethylcellulose (HPMC). PBS and 100 mg/ml IgG prior to apllication on cTMPC cultivated for 21 days at air-liquid interface (ALI) or tarcheal tissue. The video was created using a Keyence BZ-X800 microscope (Keyence. Osaka. Japan) and was uploaded to https://youtu.be/IDJPztaiaMg?si=GKhZL0M8hH8AcjeF.

Supplementary Table S5: Gene expression analysis of Fc-receptors (FCGRT. FCGR1A. FCGR2A. FCGR2B. FCGR3A). *GAPDH* was used as housekeeping gene. cTMPC: n = 2. N = 2. C: TMPC DS: n = 3. N = 3. x-fold change is shown as mean ± SD and was calculated in comparison to tracheal mucosa.

**mRNA target**	**cTMPC**	**TMPC DS**
FCGRT	0.73 ± 0.098	0.94 ± 0.368
FCGR1A	0.097 ± 0.096	0.0024 ± 0.019
FCGR2A	below detection	0.103 ± 0.026
FCGR2B	below detection	0.022 ± 0.01
FCGR3A	0.008 ± 0.0063	0.0006 ± 0.001
